# Deficiency of optineurin enhances osteoclast differentiation by attenuating the NRF2-mediated antioxidant response

**DOI:** 10.1038/s12276-021-00596-w

**Published:** 2021-04-16

**Authors:** Peng Xue, Xiangxiang Hu, Emily Chang, Lufei Wang, Minghui Chen, Tai-Hsien Wu, Dong-Joon Lee, Brian L. Foster, Henry C. Tseng, Ching-Chang Ko

**Affiliations:** 1 Oral and Craniofacial Health Science Institute, School of Dentistry, UNC at Chapel Hill, North Carolina, NC USA; 2 Division of Orthodontics, The Ohio State University College of Dentistry, Columbus, OH 43210 USA; 3 Duke Eye Center and Department of Ophthalmology, Duke University Medical Center, Durham, NC 27710 USA; 4 Division of Biosciences, The Ohio State University College of Dentistry, Columbus, OH 43210 USA

**Keywords:** Mechanisms of disease, Stress signalling

## Abstract

Abnormally increased resorption contributes to bone degenerative diseases such as Paget’s disease of bone (PDB) through unclear mechanisms. Recently, the optineurin (OPTN) gene has been implicated in PDB, and global OPTN knockout mice (*Optn*^*−/−*^) were shown to exhibit increased formation of osteoclasts (osteoclastogenesis). Growing evidence, including our own, has demonstrated that intracellular reactive oxygen species (ROS) stimulated by receptor activator of nuclear factor kappa-B ligand (RANKL) can act as signaling molecules to promote osteoclastogenesis. Here, we report that OPTN interacts with nuclear factor erythroid-derived factor 2-related factor 2 (NRF2), the master regulator of the antioxidant response, defining a pathway through which RANKL-induced ROS could be regulated for osteoclastogenesis. In this study, monocytes from *Optn*^*−/−*^ and wild-type (*Optn*^*+/+*^) mice were utilized to differentiate into osteoclasts, and both qRT-PCR and tartrate-resistant acid phosphatase (TRAP) staining showed that the *Optn*^*−/−*^ monocytes exhibited enhanced osteoclastogenesis compared to the *Optn*^*+/+*^ cells. CellROX^®^ staining, qRT-PCR, and Western blotting indicated that OPTN deficiency reduced the basal expression of *Nrf2*, inhibited the expression of NRF2-responsive antioxidants, and increased basal and RANKL-induced intracellular ROS levels, leading to enhanced osteoclastogenesis. Coimmunoprecipitation (co-IP) showed direct interaction, and immunofluorescence staining showed perinuclear colocalization of the OPTN-NRF2 granular structures during differentiation. Finally, curcumin and the other NRF2 activators attenuated the hyperactive osteoclastogenesis induced by OPTN deficiency. Collectively, our findings reveal a novel OPTN-mediated mechanism for regulating the NRF2-mediated antioxidant response in osteoclasts and extend the therapeutic potential of OPTN in the aging process resulting from ROS-triggered oxidative stress, which is associated with PDB and many other degenerative diseases.

## Bone degeneration: Identifying a damage-limiting protein

The protein optineurin is implicated in regulating the breakdown of bone during natural maintenance by a mechanism that may be relevant to understanding and treating degenerative bone diseases. Cells called osteoclasts mediate bone breakdown during natural bone turnover, but excessive breakdown occurs in some bone diseases. Working with cultured mouse cells, researchers in the USA, led by Ching-Chang Ko at Ohio State University, Columbus, and Henry Tseng at Duke University, identified a molecular signaling pathway in which optineurin interacts with a protein called NRF2, which is known as the master regulator of the antioxidant response. This interaction allows optineurin to limit the production of chemicals called reactive oxidative species which stimulate osteoclast generation. The findings suggest that drugs regulating the optineurin signaling pathway may help treat bone degeneration and possibly other degenerative diseases.

## Introduction

During skeletal remodeling, bone formation by osteoblasts is balanced with bone degradation by osteoclasts to meet the functional demands of the skeletal system^1,2^. An imbalance due to hyperactive osteoclastic activity results in net bone loss^3,4^ and abnormal remodeling, contributing to severe degenerative bone diseases such as Paget’s disease of bone (PDB), a chronic disorder most commonly affecting older people^5–8^. PDB is increasing in prevalence with the aging population and afflicts millions of individuals worldwide^9,10^. There is no cure or effective treatment for bone degeneration caused by hyperactivated osteoclastogenesis in PDB, and the underlying molecular mechanisms remain unknown.

Mature osteoclasts are derived from differentiating osteoclast precursors. This process, called osteoclastogenesis, is triggered by the binding of receptor activator of nuclear factor kappa-B ligand (RANKL) to its receptor on osteoclastic progenitors (preosteoclasts), which activate multiple downstream signaling pathways^11^, in which intracellular reactive oxidative species (ROS) have recently been demonstrated to serve as critical secondary messengers^12–15^. In contrast to physiological levels of ROS, which serve as signaling molecules^16–18^, excess ROS levels may result in abnormally elevated osteoclastogenesis, oxidative stress, and cellular damage over time^19,20^. Conversely, ROS scavengers have been consistently shown to inhibit osteoclastogenesis^21–29^. Beyond bone degeneration, ROS have also been broadly associated with age-dependent cellular degeneration in other tissues^30–32^.

Optineurin (OPTN), an adapter protein involved in numerous cellular functions, has recently been implicated in PDB^33–35^. We previously showed that aged mice genetically lacking optineurin (OPTN)^36^ exhibited hyperactivated osteoclastogenesis, focal bone lesions, and other PDB-like clinical features, while young mice did not display these phenotypes, although the mechanisms remain undefined. OPTN is expressed in bone and neuronal cells and has been reported to protect cells from ROS-induced cell damage^37,38^, suggesting a regulatory role of ROS in OPTN-mediated osteoclastogenesis. However, how OPTN regulates ROS homeostasis in osteoclasts remains unexplored.

Intracellularly, ROS homeostasis is mediated by nuclear factor-erythroid 2-related factor 2 (NRF2; also known as NFE2L2), a transcription factor that acts as a master regulator of the cellular antioxidant response. NRF2 has a basic-region leucine zipper (bZIP) domain^39^ that allows it to heterodimerize with other proteins, regulates its stability, and modulates transcriptional activity^40,41^. Interestingly, OPTN also contains a bZIP domain^42^, identifying it as a potential NRF2 binding partner that might contribute to the regulation of ROS levels and osteoclastogenesis^43^. However, the direct Nrf2 and OPTN interaction as a ROS homeostatic mechanism has not been demonstrated.

In this study, we tested the hypothesis that OPTN interacts with NRF2 to modulate RANKL-induced ROS signaling and osteoclastogenesis. Our results will provide novel insights into how loss of normal OPTN function results in attenuated antioxidant responses and elevated osteoclastogenesis. These insights will improve our understanding of abnormal bone remodeling and the development of therapies for bone diseases resulting from increased osteoclast activities. Because OPTN is also genetically associated with other degenerative diseases, such as glaucoma and amyotrophic lateral sclerosis (ALS), our study will potentially offer broader insights into the pathophysiology and treatment of various degenerative conditions.

## Materials and methods

### Chemicals

Receptor activator of nuclear factor kappa-B (NF-κB) ligand (RANKL, #462-TEC-010) and macrophage colony-stimulating factor (M-CSF, #416-ML-010) were obtained from R&D Systems (Minneapolis, MN). The RealTime-Glo™ MT Cell Viability Assay was purchased from Promega (Madison, WI). RNAzol was purchased from the Molecular Research Center (Cincinnati, OH). The iScript™ cDNA Synthesis Kit for RT-qPCR was purchased from Bio-Rad (Hercules, CA). The dye 2′,7′-dichlorofluorescin diacetate (DCF, #D6883) for ROS measurement was purchased from Sigma-Aldrich (St. Louis, MO). Dulbecco’s modified Eagle’s medium (DMEM), fetal bovine serum (FBS), penicillin, and streptomycin were all obtained from Thermo Fisher Scientific (Waltham, MA). The NRF2 inducers curcumin (CUR, #C1386), quercetin (QUE, #Q4951), bardoxolone methyl (CDDO-Me, #SMB00376), sulforaphane (SFN, #S4441), and tert-butylhydroquinone (tBHQ, #112941) were all purchased from Sigma-Aldrich.

### Animals

Global *Optn* knockout (*Optn*^−/−^) mice on a C57BL/6 background were generated by crossing *Optn*^*flox/flox*^ mice with *CMV-Cre* mice (Jackson Laboratories, Bar Harbor, ME) as described in our previous study^36^. All animal procedures were approved by the Institutional Animal Care and Use Committees at the University of North Carolina at Chapel Hill and Duke University.

### Cell culture

For primary culture of osteoclast precursors, 8- to 12-week-old *Optn*^−/−^ and *Optn*^+/+^ mice were euthanized by CO_2_, and tibias and femurs were collected. Bone marrow cells were flushed out into α-MEM medium containing 10% FBS and penicillin/streptomycin and cultured at 37°C in a humidified 5% CO_2_ atmosphere. After one day, nonadherent cells were collected and reseeded in plates with 30 ng/mL M-CSF for 3 days to grow osteoclast precursor cells. Cycloheximide (0.5 mM, C7698-1G, Sigma-Aldrich) was used to inhibit protein synthesis.

### Osteoclast differentiation and TRAP staining

For osteoclast differentiation, osteoclast precursors in 48-well plates were treated with RANKL (10 ng/mL) and M-CSF (30 ng/mL) for 6 days. The cells were fixed with 10% paraformaldehyde for 10 min before incubation in TRAP staining solution for 10–30 min to detect TRAP^+^ multinucleated osteoclast-like cells. TRAP-stained plates were scanned and imaged using an Eclipse Ti microscope (Nikon, Shinagawa, Japan).

### Cell viability assay

Cell viability was measured by the RealTime-Glo™ MT Cell Viability Assay according to the manufacturer’s directions (Promega). In brief, a cell suspension was prepared by adding MT Cell Viability reagents to the culture media. Cells were seeded in this medium and cultured for up to 3 days. The luminescence of the cells was measured via a Cytation 5 plate reader (BioTek, Winooski, VT). Cell viability is expressed as the relative fold change compared to that of control cells cultured in normal growth medium.

### Measurement of intracellular ROS

Qualitative and quantitative analyses of intracellular ROS in compound-treated cells were performed by fluorescence microscopy using CellROX Green/Nucblue staining reagents. ROS levels were also assessed by a plate reader using 2′,7′-dichlorofluorescin diacetate (DCF) as previously described^29^. In brief, osteoclast precursors were incubated with 10 μM DCF for 15 min at 37°C, rinsed in PBS three times, stimulated with RANKL (100 ng/mL) for up to 15 min and lysed with Tris-HCl (0.01 M)/0.5% Triton X-100 (pH 7.4). Fluorescence of the lysate was measured by a plate reader at 485 nm excitation and 535 nm emission.

### RT-qPCR

Total mRNA of osteoclast precursor cells was extracted using RNAzol and reverse-transcribed using the iScript™ Kit. Subsequently, PCR reactions were prepared by using iTaq^TM^ Universal SYBR Green Supermix and performed on a StepOnePlus Real-time PCR system (Applied Biosystems). Primers for target genes are listed in Supplementary Table S1. Threshold cycles of primers were normalized to the housekeeping gene β-actin, and the relative values were calculated based on the comparative Ct method (2^−ΔΔCt^ method) as previously described^29^.

### Western blot

Protein samples were prepared either by RIPA buffer (R0278, Sigma-Aldrich) to yield total protein or by a nuclear extraction kit (Cat. #2900, Millipore Sigma) to yield cytoplasmic and nuclear proteins. The Criterion Vertical Electrophoresis Cell and Trans-Blot Turbo Transfer System (Bio-Rad) was used for immunoblot analysis as described in our previous study^29^. Protein expression was detected by ECL Prime Western Blotting Detection Reagent (GE Healthcare Amersham). Primary antibodies used in the study were against NRF2 (#1272 T, Cell Signaling Technology), p-NRF2 Ser40 (#PA5-67520, Invitrogen), KEAP1 (#sc-514914, Santa Cruz Biotechnology), HMOX1 (#SC-136960, Santa Cruz Biotechnology), NQO1 (#sc-32793, Santa Cruz Biotechnology) and β-ACTIN (#SC-47778, Santa Cruz Biotechnology). HRP-linked anti-rabbit or anti-mouse IgG antibodies (#7074P2, #7076P2, Cell Signaling Technology) were used as secondary antibodies.

### In vitro coimmunoprecipitation assays

The NC16 pCDNA3.1 FLAG NRF2 plasmid and pDEST26-OPTN plasmid were purchased from Addgene (Watertown, MA). HEK293T cells were seeded in 6-well plates and transfected with the indicated plasmids at a concentration of 2000 ng per plasmid/well by Lipofectamine 2000 according to the manufacturer’s protocol. Transfected cells were lysed in NP-40 buffer and split into two groups: 200 µl fractions for the input assay and 800 µl fractions for coimmunoprecipitation. The input lysates were resolved on SDS-PAGE gels and analyzed by immunoblot. The coimmunoprecipitated lysates were incubated with 20 µl of Anti-Flag^®^ M2 Affinity Gel (Millipore Sigma A2220) for 24 h. After incubation, the samples were centrifuged at 5000 × *g* for 1 min, and the pellets were washed five times with NP-40 buffer. The proteins were finally resolved on SDS-PAGE gels and analyzed by immunoblot.

### Confocal microscopy

*Optn*^−/−^ and *Optn*^+/+^ preosteoclasts were fixed with 4% paraformaldehyde for 24 h. After three washes with PBS, the cells were permeabilized with 0.3% Triton X-100 for 10 min and washed three times again with PBS. After the cells were blocked with horse serum for 24 h, they were incubated with anti-rabbit OPTN (Abcam, ab23666, 1:150) and anti-rat NRF2 (Cell Signaling, #14596, 1:150) primary antibodies; anti-rat NRF2 (Cell Signaling, #14596, 1:150) and anti-mouse KEAP1 (Santa Cruz Biotechnology, #sc-514914, 1:50) primary antibodies; anti-mouse KEAP1 (Santa Cruz Biotechnology, #sc-514914, 1:50) and anti-rabbit SQSTM1/p62 (Abcam, ab240635, 1:150) primary antibodies overnight at 4°C. The cells were then incubated with goat anti-rabbit Alexa Fluor 488 (Thermo Fisher, #A32731, 1:200), goat anti-rat Alexa Fluor 594 secondary antibodies (Thermo Fisher, #A11007, 1:200), goat anti-mouse Alexa Fluor 488 (Thermo Fisher, #A28175, 1:200), and goat anti-mouse Alexa Fluor 594 (Thermo Fisher, #A11032, 1:200) accordingly for 2 h at room temperature. After DAPI staining, the cells were imaged by confocal microscopy using the FITC, CY3, and DAPI channels.

### Image quantification

For protein quantification by western blotting, ImageJ (version 1.52k; Bethesda, MD) was used to analyze the gray value of each band to represent intensity, which was normalized to β-actin. For intracellular ROS quantification, ImageJ was applied to measure the area integrated intensity of the ROS fluorescence signal in each cell, and a total of 15 cells were randomly contoured and calculated in each image. The mean value of ROS fluorescence represented the intensity of each image. Each analysis was performed three times to determine statistical significance. For quantification of translocated NRF2, ImageJ was applied to identify the NRF2^±^DAPI^±^ area (region of interest (ROI)) shown in white (Supplementary Fig. S2c). The mean fluorescence value of the ROI in each nucleus represents the intensity of translocated NRF2 per nucleus in each image. Each analysis was performed three times to determine statistical significance.

### Statistical analyses

Statistical analyses were performed using Prism 5 (GraphPad Software, San Diego, CA). Significance was determined as *p* < 0.05. Comparisons with a specific control were assessed using one-way analysis of variance (ANOVA) followed by the Bonferroni t-test. Data are expressed as the mean ± standard error of the mean (SEM).

## Results

### OPTN deficiency enhances osteoclastogenesis

**Fig. 1:**
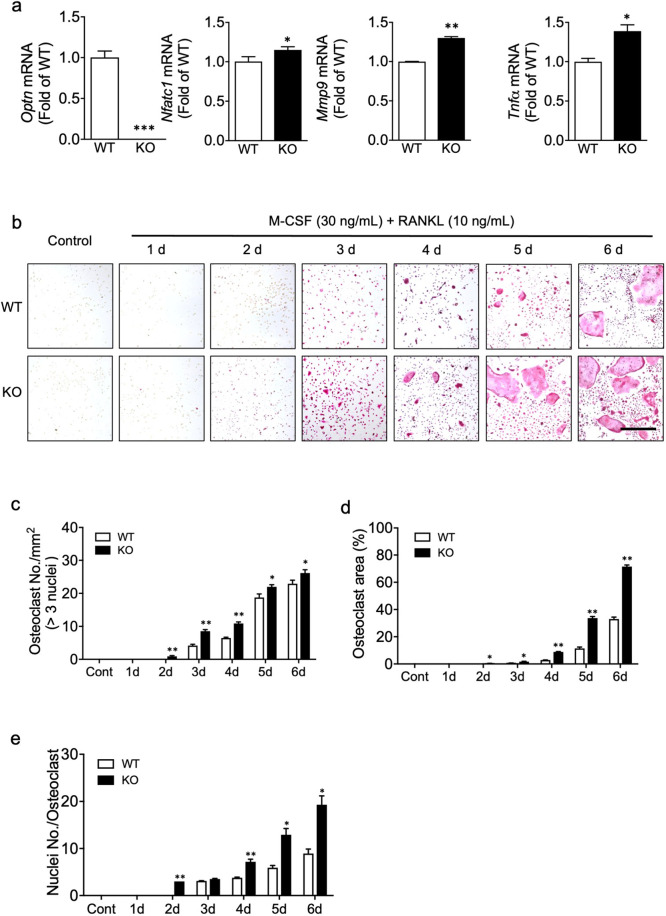
OPTN deficiency enhances osteoclastogenesis. **a** Higher expression of key osteoclast marker genes in OPTN KO vs. *Optn*^*+/+*^ preosteoclast precursors under basal, unstimulated conditions. **b** Formation of mature osteoclasts from precursors isolated from the *Optn*^*+/+*^ and *Optn*^*−/−*^ mice. Primary osteoclast precursors were treated with RANKL (10 ng/mL) and M-CSF (30 ng/mL) for 6 days. Mature osteoclasts appear as multinucleated TRAP^+^ (red) cells. Representative images are shown in the panel. Scale bar = 500 μm. **c** Osteoclast number per mm^2^, **d** the percentage of osteoclast area per image, and **e** the number of nuclei per osteoclast. WT, *Optn*^*+/+*^; KO, *Optn*^*−/−*^; d, day. Control (growth media only). *n* = 3 experiments. Data are presented as the mean ± SEM. *p < 0.05, **p < 0.01, ***p < 0.001 compared to *Optn*^*+/+*^ within each group.

To test the effects of OPTN ablation on osteoclastogenesis, we determined the gene expression of *Optn* and osteoclast markers in osteoclast precursors under basal conditions (macrophages treated with M-CSF only). As expected, *Optn* transcripts were undetectable in the *Optn*^*−/−*^ vs. *Optn*^*+/+*^ osteoclast precursors (Fig. 1a). In addition, the expression of several key osteoclastogenic markers, including *Nfatc1*, *Mmp9*, and *Tnfα*, was significantly increased in the *Optn*^*−/−*^ vs. *Optn*^*+/+*^ macrophages, supporting a role for OPTN in osteoclast differentiation.

To further investigate the role of OPTN, we treated the *Optn*^*+/+*^ and *Optn*^*−/−*^ precursor cells with RANKL and M-CSF for 6 days to promote osteoclast differentiation, where precursors differentiate and merge into multinucleated giant TRAP^+^ cells that represent mature osteoclasts. Significantly enhanced osteoclastogenesis was observed in the *Optn*^*−/−*^ vs. *Optn*^*+/+*^ cells (Fig. 1b), based on more mature osteoclasts per area (Fig. 1c), larger average osteoclast size (Fig. 1d), and more nuclei per cell (Fig. 1e). These results identify OPTN as a negative regulator of osteoclastogenesis.

### OPTN deficiency results in elevated ROS production in preosteoclasts and osteoclasts

**Fig. 2:**
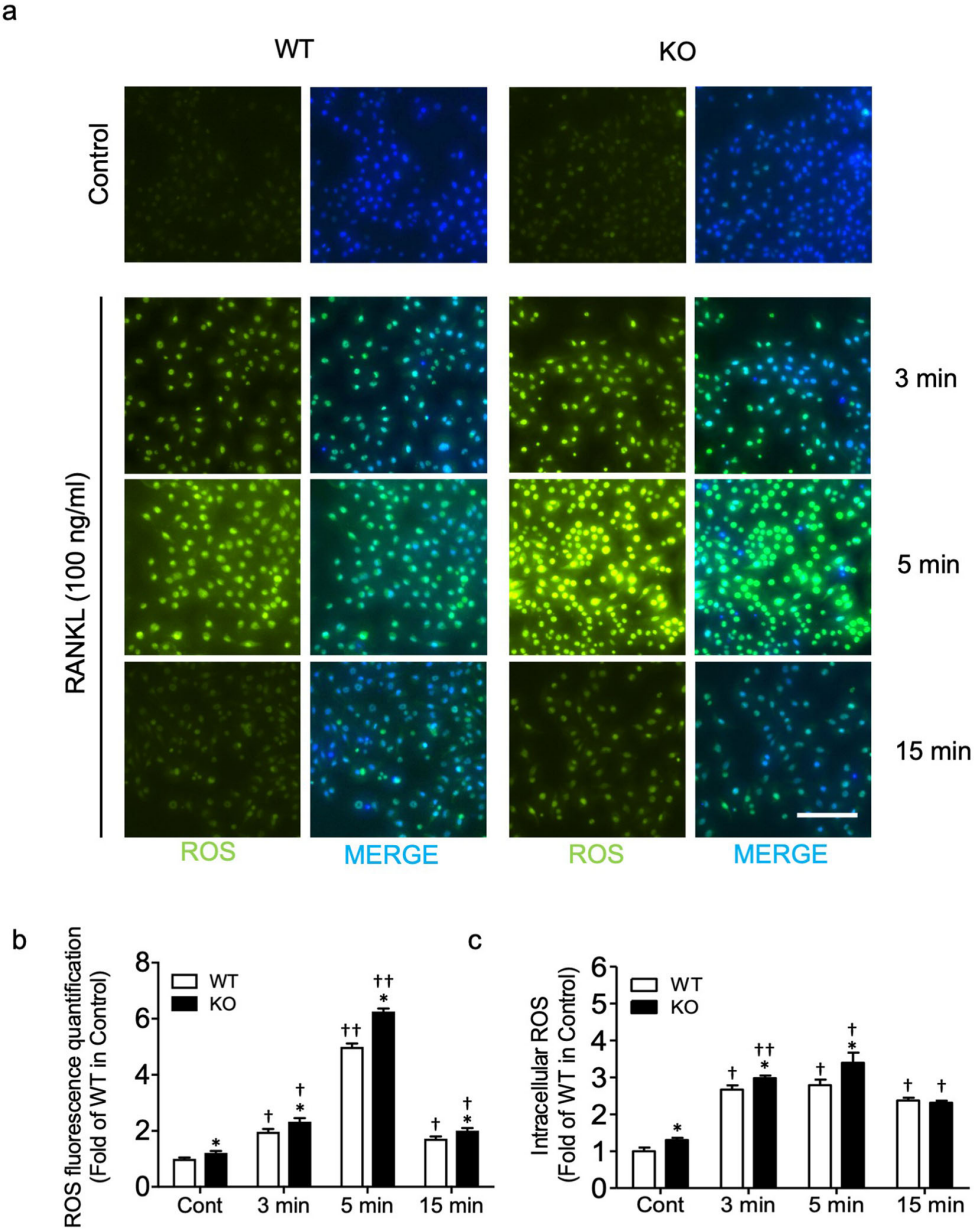
OPTN deficiency results in elevated ROS in preosteoclasts and osteoclasts. **a** Merged images of CellROX Green fluorescence and DAPI staining in osteoclast precursors treated with RANKL (100 ng/mL) for the indicated time points (3, 5, and 15 min). Scale bar = 200 μm. **b** Quantification of CellROX Green fluorescence. **c** The intracellular ROS levels induced by RANKL (100 ng/mL) were determined using DCF staining and a plate reader. WT, *Optn*^*+/+*^; KO, OPTN-knockout; Cont, control (growth media only); *n* = 3 experiments. Data are presented as the mean ± SEM. *p < 0.05, **p < 0.01 compared to *Optn*^*+/+*^ within each time point group; ^†^*p* < 0.05, ^††^*p* < 0.01 compared to the untreated control of either the *Optn*^*+/+*^ or *Optn*^*−/−*^ group.

Among the various pathways that participate in osteoclastogenesis, ROS have been reported to act as secondary messengers during RANKL-induced osteoclast differentiation^15,23,26,28,29,44^. To further elucidate the role of ROS in OPTN-mediated osteoclastogenesis, we treated *Optn*^*+/+*^ and *Optn*^−/−^ preosteoclasts with a high concentration of RANKL (100 ng/mL), which is reported to trigger downstream ROS signaling^15,28,29^, and assessed basal and RANKL-induced intracellular ROS levels by CellROX Green staining and fluorescence microscopy. In cells without RANKL treatment, we observed a higher level of basal ROS in the *Optn*^*−/−*^ vs. *Optn*^*+/+*^ cells (Fig. 2a, first row). RANKL treatment induced significantly higher levels of ROS in both the *Optn*^*−/−*^ and *Optn*^*+/+*^ cells than the control cells. Furthermore, the ROS level increased by 3 min, reached its peak at 5 min, and declined by 15 min (Fig. 2a, b), a pattern consistent with a previous report^14^. Since ROS are highly reactive and unstable, an additional quantitative analysis by DCF staining for intracellular ROS successfully confirmed these results (Fig. 2c). These findings revealed that loss of OPTN contributes to elevated intracellular ROS in both basal and RANKL-stimulated conditions.

### Optn^*−/−*^ cells are more sensitive to H_2_O_2_-induced cytotoxicity than Optn^+/+^ cells

**Fig. 3:**
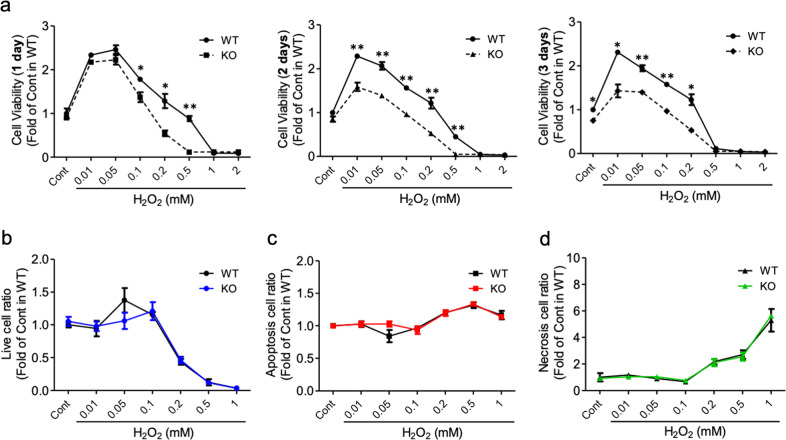
Optn^*−/−*^ cells are more sensitive to H2O2-induced cytotoxicity. **a** Cell viability of osteoclast precursors in response to various concentrations of H_2_O_2._ Each graph indicates a different treatment duration (1, 2, and 3 days). Quantitation of live **b**, apoptotic **c**, and necrotic **d** cells following treatment with various concentrations of H_2_O_2_. The cells are shown as a ratio of treated versus control untreated cells. The cells were treated with H_2_O_2_ for 1 day and then stained with Annexin V for apoptosis and necrosis before quantitation by flow cytometry. WT, *Optn*^*+/+*^ preosteoclasts; KO, *Optn*^*−/−*^ preosteoclasts; Cont, control (growth media only); *n* = 3 experiments. Data are presented as the mean ± SEM. *p < 0.05, **p < 0.01 compared to *Optn*^*+/+*^ within each dosage group.

Notably, ROS levels in the *Optn*^*−/−*^ cells were significantly higher than those in the *Optn*^*+/+*^ cells even under basal conditions, suggesting that the redox state in OPTN-deficient cells was affected (Fig. 2a, b). To further examine the antioxidant response in the *Optn*^*−/−*^ cells, we treated these cells with H_2_O_2_, an oxidative stressor that promotes cellular oxidative damage. Using the MT cell viability assay to assess cell viability, we observed that both the *Optn*^*+/+*^ and *Optn*^*−/−*^ cells were sensitive to H_2_O_2_ in a concentration-dependent manner. However, within the range of 0.01 to 1 mM, the *Optn*^*−/−*^ cells were more susceptible to H_2_O_2_ than the *Optn*^*+/+*^ cells (Fig. 3a). Beyond 1 mM H_2_O_2_ exposure, cells with both genotypes showed complete cell death. In a time-course study, the *Optn*^*−/−*^ cells were more sensitive than the *Optn*^*+/+*^cells to H_2_O_2_ exposure at 1, 2, and 3 days (Fig. 3a). To evaluate whether H_2_O_2_ caused apoptosis or necrosis in cells, we performed annexin-PI staining (Fig. 3b, c, d). Significant cell death began after 0.1 mM H_2_O_2_ treatment, consistent with the cell viability results. Furthermore, cell death was necrotic and not apoptotic for both genotypes, and no significant difference was observed between the two groups of cells for necrotic cell death. Taken together, our results showed that OPTN deficiency in preosteoclasts could increase cellular sensitivity to H_2_O_2_-induced cytotoxicity but primarily affects cell viability and not cell death.

### Deficiency of OPTN attenuates Nrf2 and antioxidant expression

**Fig. 4:**
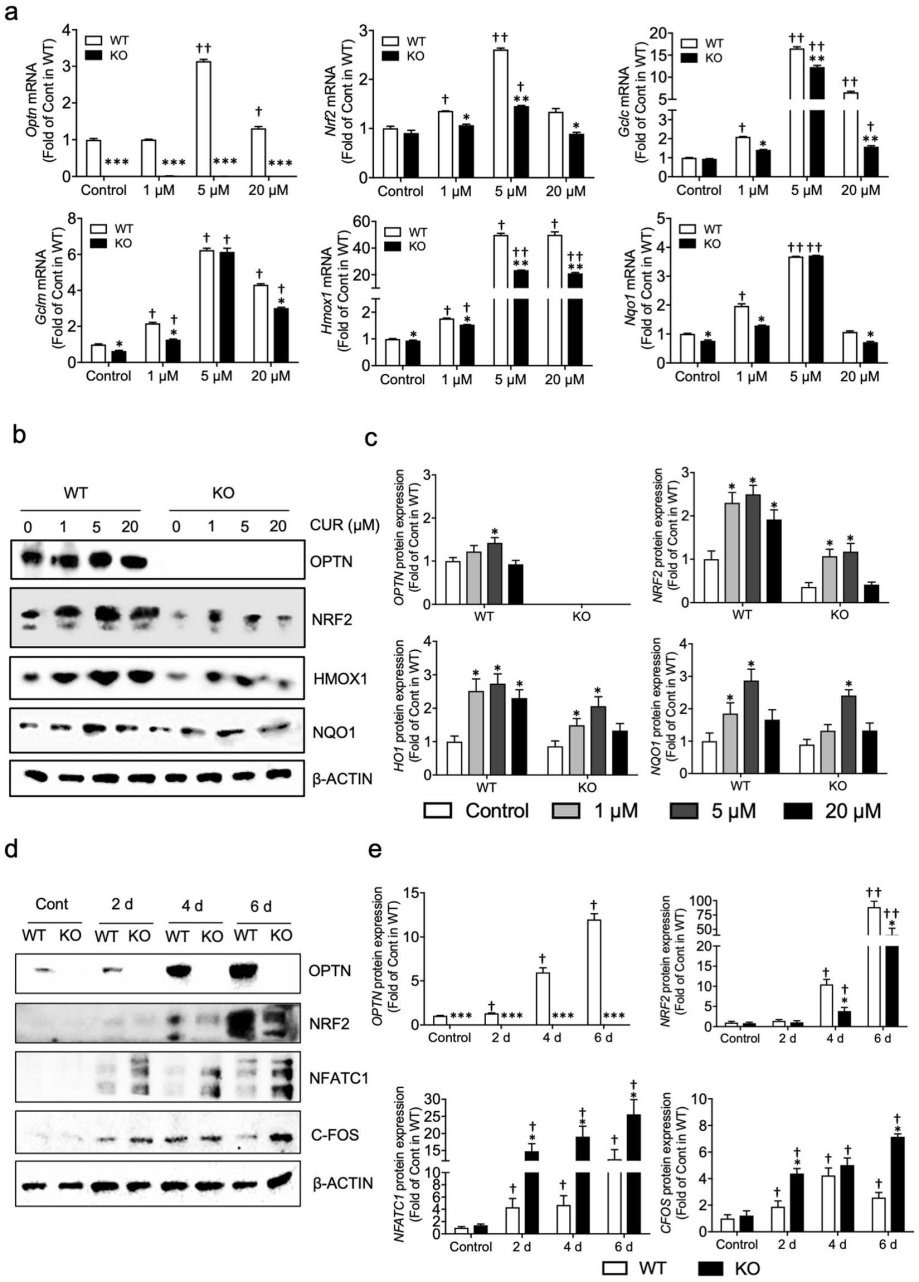
Deficiency of OPTN attenuates Nrf2 and antioxidant expression. **a** Decreased gene expression of *Nrf2* and *Nrf2* antioxidants in the curcumin-treated *Optn*^*−/−*^ vs. *Optn*^*+/+*^ osteoclast precursors. Cells were treated with curcumin (1, 5 and 20 µM) for 6 h. Gene expression of the NRF2-mediated antioxidant genes *Hmox1*, *Gclc*, *Gclm*, and *Nqo1* was determined by RT-qPCR with all results were normalized with β-actin. **b** Decreased protein expression of NRF2 and regulated antioxidants in the curcumin-treated *Optn*^*+/+*^ and *Optn*^*−/−*^ preosteoclasts. The cells were treated with curcumin (1, 5 and 20 µM) for 6 h, whole proteins were extracted, and the OPTN, NRF2, HMOX1, and NQO1 levels were measured. **c** Quantification of WB bands for the protein expression of Nrf2-mediated antioxidants in the curcumin-treated *Optn*^*+/+*^ and *Optn*^*−/−*^ preosteoclasts. **d** Decreased protein levels of NRF2 and key osteoclastogenic markers during osteoclast differentiation in the *Optn*^*−/−*^ cells. Primary osteoclast precursors were treated with RANKL (10 ng/ml) and M-CSF (30 ng/mL) for 6 days. At the indicated time points (2, 4, and 6 d), whole protein extracts of the *Optn*^*+/+*^ and *Optn*^*−/−*^ cells were extracted and analyzed by Western blotting. **e** Quantification of WB bands for the protein levels of NRF2 and key osteoclastogenic markers during osteoclast differentiation. CONT, control (growth media only); *n* = 3 experiments. Data are presented as the mean ± SEM. (a): *p < 0.05, **p < 0.01, ***p < 0.001 compared to *Optn*^*+/+*^ within each dosage group; ^†^*p* < 0.05, ^††^*p* < 0.01 compared to the untreated control of either the *Optn*^*+/+*^ or *Optn*^*−/−*^ group; (**c**): *p < 0.05 compared to the untreated control of either the *Optn*^*+/+*^ or *Optn*^*−/−*^ group; **e**: *p < 0.05, **p < 0.01, ***p < 0.001 compared to *Optn*^*+/+*^ within each time point group; ^†^*p* < 0.05, ^††^*p* < 0.01 compared to the untreated control of either the *Optn*^*+/+*^ or *Optn*^*−/−*^ group.

The results described above suggest that the *Optn*^*−/−*^ cells are more sensitive to oxidative damage induced by H_2_O_2_, indicating impairment of the antioxidative system in these cells. Among the molecules involved in the antioxidant response, NRF2 is considered to be a master transcription factor that can trigger downstream antioxidants to scavenge excess intracellular ROS^26,29,45,46^. Thus, we next investigated how OPTN deficiency alters antioxidant capability in preosteoclasts. Antioxidant gene expression was first assessed in the *Optn*^*−/−*^ and *Optn*^*+/+*^ cells in response to curcumin, a well-known activator of NRF2^28,47^, at concentrations of 1, 5, or 20 µM. As expected, we found no OPTN expression in the *Optn*^*−/−*^ cells. Surprisingly, 5 and 20 µM curcumin induced OPTN expression in the *Optn*^*+/+*^ cells (Fig. 4a).

At basal levels, *Nrf2* expression trended lower in the *Optn*^*−/−*^ vs. *Optn*^*+/+*^ cells, while the mRNA level was significantly decreased in the *Optn*^*−/−*^ vs. *Optn*^*+/+*^ cells for the NRF2-mediated antioxidants *Gclc*, *Gclm*, *Hmox1*, and *Nqo1*. A similar pattern of decreased antioxidant gene expression was also observed in both liver tissues and bone marrow myeloid cells from the *Optn*^*−/−*^ animals (Supplementary Fig. S1). Under the treatment conditions used here, Nrf2 was induced by curcumin in the *Optn*^*+/+*^ cells and showed a peak of expression at 5 µM curcumin but was induced to a lesser extent in the *Optn*^*−/−*^ cells. With respect to NRF2-mediated antioxidants, expression was slightly increased in both genotypes when exposed to 1 µM curcumin, while the *Optn*^*−/−*^ cells exhibited lower antioxidant expression. In the 5 µM curcumin treatment group, all the antioxidants were significantly increased in both the *Optn*^*+/+*^ and *Optn*^*−/−*^ cells compared to the controls. However, the *Hmox-1* and *Gclc* levels were significantly lower in the *Optn*^*−/−*^ cells than in the *Optn*^*+/+*^ cells, while the *Gclm* and *Nqo1* levels showed a decreasing trend (Fig. 4a). Similarly, in the 20 µM curcumin treatment group, significantly lower antioxidant expression was observed in the *Optn*^*−/−*^ cells than in the *Optn*^*+/+*^ cells.

We next assessed the protein expression of NRF2 and its mediated antioxidants. Western blot analysis revealed a similar trend in that the NRF2, HMOX1, and NQO1 levels were lower in the *Optn*^*−/−*^ than *Optn*^*+/+*^ cells under basal or/and curcumin-treated conditions (Fig. 4b, c). Although phosphorylation of NRF2 at multiple sites has a limited contribution to modulating the Nrf2-dependent antioxidant response^48^, we tested this possibility by performing Western blotting for phosphorylated NRF2 (p-NRF2). There was no significant difference in the expression of p-NRF2 at Ser40 between wild-type OPTN^*−/−*^ preosteoclasts or mature osteoclasts (Supplementary Fig. S2a). These results suggested that OPTN deficiency downregulates NRF2 expression, results in a lack of phosphorylation of NRF2, and attenuates the expression of Nrf2-induced antioxidants. To further evaluate whether NRF2 participates in the process of osteoclastogenesis, we measured the NRF2 protein levels in both the *Optn*^*−/−*^ and *Optn*^*+/+*^ cells during the differentiation of precursors into mature osteoclasts (Fig. 4d, e). OPTN protein increased during osteoclastogenesis in the *Optn*^*+/+*^ cells, and while the NRF2 protein levels increased in both cell types, expression in the *Optn*^*−/−*^ cells was significantly lower than that in the *Optn*^*+/+*^ cells. Both the gene and protein levels of antioxidants were also decreased (Supplementary Fig. S2a, c). These findings were observed in preosteoclasts during differentiation into mature osteoclasts. The expression of osteoclastogenic markers, including NFATC1 and C-FOS, was elevated in the *Optn*^*−/−*^ cells compared to the *Optn*^*+/+*^ cells during osteoclastogenesis, consistent with TRAP staining findings (Fig. 1b). These collective data strongly indicated that OPTN deficiency enhances osteoclastogenesis via a decreased NRF2-mediated antioxidant response, which plays a critical regulatory role in ROS signaling.

### Direct interaction between the NRF2 and OPTN proteins

**Fig. 5:**
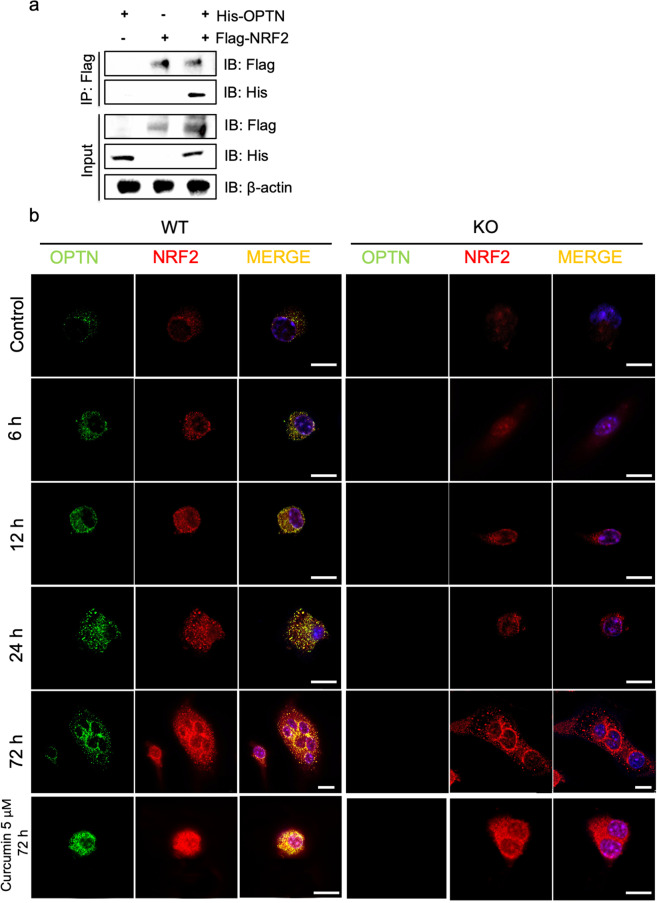
Direct interaction between the NRF2 and OPTN proteins. **a** Immunoblot analysis of whole-cell lysates and CoIP assays using HEK293T cells. Flag immunoprecipitates were isolated from HEK293T cells transfected with His-OPTN or Flag-Nrf2 plasmids for 1 d. The IP bands showed that Flag-tagged NRF2 can pull down His-tagged OPTN, indicating that NRF2 and OPTN can bind with each other in vitro. β-Actin expression served as a loading control. **b** Intracellular localization of OPTN and NRF2 in preosteoclasts. The *Optn*^*−/−*^ and *Optn*^*+/+*^ preosteoclasts were treated with M-CSF (10 ng/mL) and RANKL (30 ng/mL) for 6, 12, 24, and 72 h. RANKL + 5 μM curcumin treatment for 72 h was used as a positive control. After fixation, the cells were processed with immunofluorescence using antibodies against OPTN (green), NRF2 (red) and nuclei (blue). In merged images, colocalization of OPTN and NRF2 was observed mostly in perinuclear granular structures (yellow). Scale bar = 10 μm.

To investigate whether OPTN directly interacts with NRF2, we performed an immunoprecipitation (IP) assay using HEK293T cells. After transient overexpression of FLAG-tagged NRF2 and His-tagged OPTN in cells, we pulled down His-OPTN by anti-FLAG antibodies, suggesting that OPTN and NRF2 can directly interact with each other (Fig. 5a). Both OPTN and NRF2 have been previously identified in the cytoplasm and nucleus^36,40,49^, and as such, there will be two potential subcellular locations for the OPTN-NRF2 interaction: 1) OPTN may bind to NRF2 in the cytoplasm to facilitate NRF2 translocation or to maintain NRF2 stability against proteasomal degradation^50^ or 2) OPTN could translocate to the nucleus and bind to NRF2 and other binding partners to form a complex that triggers antioxidant expression.

To examine the two possibilities, we visualized the localization of OPTN and NRF2 protein-protein interactions within the cell using immunofluorescence and confocal microscopy. Cells were stimulated with RANKL to initiate osteoclastogenesis, followed by endogenous NRF2 and OPTN immunostaining at various time points (6, 12, 24, and 72 h) after stimulation. The OPTN and NRF2 binding proteins largely localized to the perinuclear region only in the *Optn*^*+/+*^ cells over time. (Fig. 5b). Surprisingly, we also observed a minor degree of colocalization of OPTN and NRF2 in the nucleus, suggesting that a small amount of nuclear OPTN could bind to NRF2, which is consistent with previous reports that OPTN was detected in the nucleus under either stress or overexpression conditions^42,49^. However, our results indicated that the amount of OPTN- and NRF2-binding proteins in the nucleus was much lower than that in the cytoplasm, indicating that the cytoplasmic interaction between OPTN and NRF2 is the likely dominant binding pattern between OPTN and NRF2 in preosteoclasts during osteoclastogenesis. To our knowledge, this is the first report that OPTN can directly interact with NRF2 predominantly in the perinuclear region in preosteoclasts.

To further elucidate these two possibilities, we performed immunoblotting for NRF2 after separating cytoplasmic and nuclear lysate fractions. We found that NRF2 was decreased in both the cytoplasm and nuclei of the *Optn*^*−/−*^ cells. Moreover, the expression of NRF2 in the *Optn*^*−/−*^ cells was reduced in nuclear lysates compared to cytoplasmic lysates (Supplementary Fig. S2b). Costaining and quantification of NRF2 and DAPI in osteoclasts also confirmed that nuclear translocation and nuclear expression of NRF2 were significantly lower in the *Optn*^*−/−*^ cells during osteoclastogenesis (Supplementary Fig. S2c, d). We then examined NRF2 degradation. We found no difference between the two types of cells in terms of NRF2 degradation following cycloheximide (protein synthesis inhibitor) treatment during osteoclastogenesis (Supplementary Fig. S3c). The Nrf2 Keap1 signaling pathway leads to degradation of NRF2 by the NRF2-KEAP1 interaction, while the noncanonical NRF2-P62 interaction leads to degradation of KEAP1 to allow NRF2 translocation^51^. We found no difference in KEAP1 expression (Supplementary Fig. S2a) or the NRF2-KEAP1 or NRF2-P62 protein-protein interactions (Supplementary Fig. S3a, b) between the two types of osteoclasts. Collectively, these data showed that OPTN is not involved in NRF2 degradation. Finally, we found no difference in the transcriptional activity of NRF2 between the two types of cells (Supplementary Fig. S3d). In general, our data suggested that OPTN binds to NRF2 in the cytoplasm and may facilitate its nuclear translocation but does not disturb KEAP1-mediated NRF2 degradation and NRF2 transcriptional activity.

### NRF2 activators attenuate increased osteoclastogenesis associated with OPTN deficiency

**Fig. 6:**
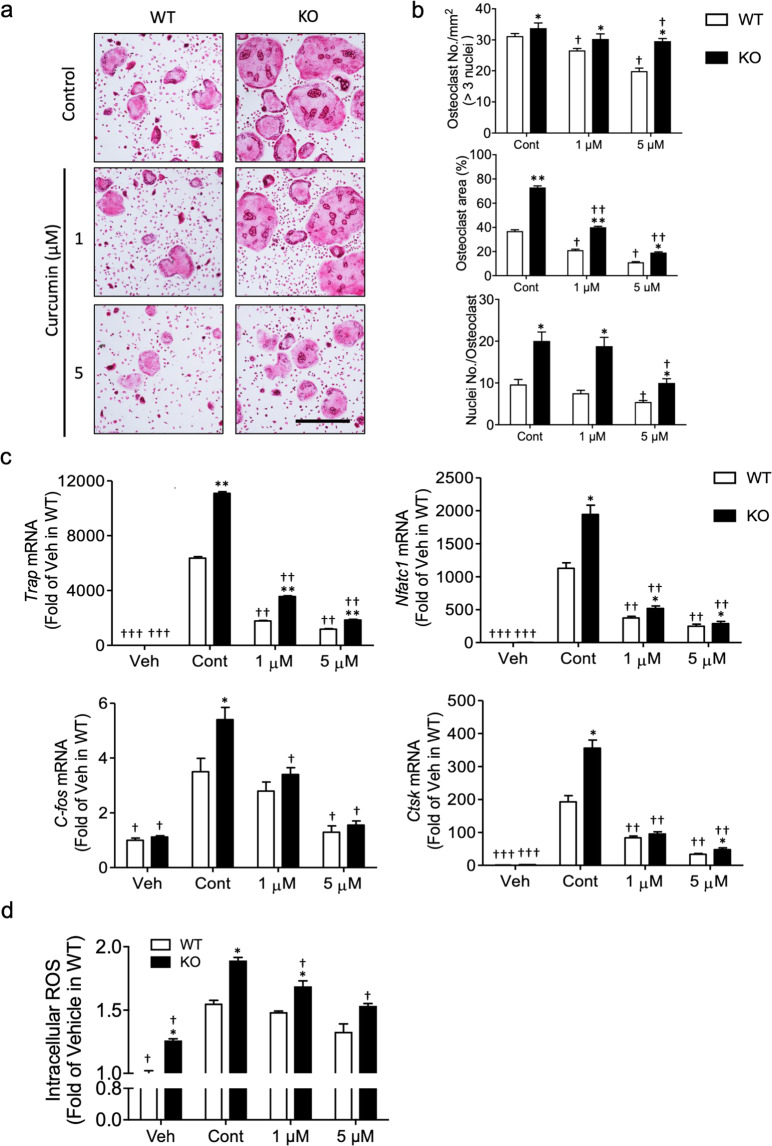
NRF2 activators attenuate increased osteoclastogenesis associated with OPTN deficiency. **a** Osteoclastogenesis is enhanced in the *Optn*^*−/−*^ cells but is reversed by curcumin treatment of osteoclast precursors. Cells were treated with RANKL/M-CSF together with various concentrations of curcumin (1 and 5 µM) for 5 days. Mature osteoclasts were identified by TRAP staining (red). Scale bar = 500 µm. **b** Osteoclast number per mm^2^, the percentage of osteoclast area per image, and nucleus number per osteoclast were quantified based on the images of Fig. 6A. **c** Osteoclastogenic gene expression in the curcumin-treated preosteoclasts. **d** Curcumin decreases intracellular ROS levels in the RANKL-treated preosteoclasts. *n* = 3 experiments. *p < 0.05, **p < 0.01, ***p < 0.001 compared to *Optn*^*+/+*^ within each dosage group; ^†^*p* < 0.05, ^††^*p* < 0.01, ^†††^*p* < 0.001 compared to the untreated control of either the *Optn*^*+/+*^ or *Optn*^*−/−*^ group.

The enhanced antioxidant expression in the *Optn*^*−/−*^ cells due to curcumin treatment (Fig. 4a, b) shows the potential for therapeutic applications, suggesting that treatment with NRF2 activators might attenuate elevated osteoclastogenesis induced by OPTN deficiency. To test this possibility, we treated the *Optn*^*−/−*^ and *Optn*^*+/+*^ cells with low (1 µM) and high (5 µM) concentrations of curcumin during osteoclastogenesis. Curcumin exhibited potent inhibition of increased osteoclastogenesis in the *Optn*^*−/−*^ cells (Fig. 6a), reduced osteoclast number per area, osteoclast size, and number of nuclei per cell in both *Optn*^*+/+*^ and *Optn*^*−/−*^ cells, and attenuated elevated osteoclast characteristics in the *Optn*^*−/−*^ vs. *Optn*^*+/+*^ cells (Fig. 6b). Curcumin treatment dramatically decreased the expression of critical osteoclastogenic markers, including *Trap*, *Nfatc1*, *C-fos*, and *Ctsk*, in both the *Optn*^*+/+*^ and *Optn*^*−/−*^ cells at the terminal stage of osteoclastogenesis (Fig. 6c). In addition to curcumin, several other NRF2 activators, including quercetin, CDDO-Me, sulforaphane, and tBHQ, showed similar effects on osteoclastogenesis (Supplementary Fig. S4).

To further elucidate the inhibitory effects of curcumin on osteoclast differentiation, we measured intracellular ROS levels upon curcumin application at the early stage of osteoclastogenesis and found that curcumin significantly attenuated RANKL-stimulated ROS elevation in both the *Optn*^*+/+*^ and *Optn*^*−/−*^ cells (Fig. 6d), suggesting that curcumin activated the NRF2-mediated antioxidant response that in turn blocked ROS signaling by scavenging RANKL-induced intracellular ROS. ROS levels in the *Optn*^*−/−*^ cells were significantly higher than those in the *Optn*^*+/+*^ cells in all the treatment groups, probably due to the reduced basal antioxidative capability in *Optn*^*−/−*^ cells. Taken together, these results suggest that curcumin and other NRF2 activators represent candidates for the treatment of OPTN-associated PDB and demonstrate that NRF2 plays a critical role in OPTN deficiency-induced hyperactive osteoclastogenesis.

## Discussion

**Fig. 7:**
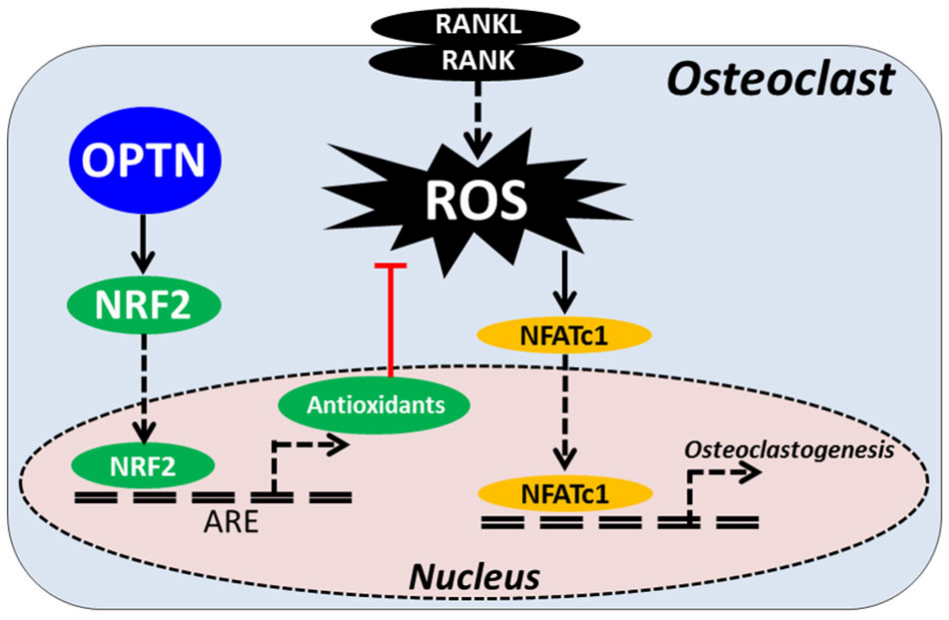
Model of hypothesized OPTN regulation of NRF2, ROS, and osteoclastogenesis. RANKL treatment generates ROS secondary messengers that activate NFATc1 and other genes involved in osteoclastogenesis. ROS homeostasis, including ROS signaling, is maintained by antioxidants, which are in turn transcriptionally regulated by NRF2 and modulated by OPTN.

Optineurin (OPTN) has been recently implicated in the differentiation of osteoclasts; however, the mechanisms identified to date by which OPTN deficiency results in elevated osteoclastogenesis have been limited to the NF-kB and interferon signaling pathways^36,52^. The different approaches to achieve OPTN deletion in animal models in these studies also contribute to confusion about the relevant underlying mechanisms. Our previous study showed that global deletion of OPTN in mice altered type I interferon signaling but not NF-κB signaling, yielding in vivo features, including osteolytic lesions that result from enhanced osteoclast differentiation and activity^36^. In this study, we identified an alternative molecular mechanism in which OPTN acts as a binding partner of NRF2, a transcription factor that modulates ROS homeostasis by regulating antioxidant expression. We showed that OPTN modulates the antioxidant response in osteoclasts, demonstrating for the first time that OPTN acts as a negative regulator of osteoclastogenesis by directly interacting with NRF2 (Fig. 7). Importantly, we showed that antioxidant treatment could attenuate OPTN deficiency-associated hyperactive osteoclastogenesis, suggesting to therapeutic possibilities of targeting this pathway.

Our discovery of a direct interaction between OPTN and NRF2 was based on reports that both OPTN and NRF2 contain a basic domain leucine zipper (bZIP) domain, a key feature of transcription factors that are capable of mediating dimerization, transcriptional regulation, and DNA binding^53^. This finding is consistent with a recent study that showed that OPTN can interact with the bZIP transcription factor neural retina leucine zipper (NRL) in the nucleus of HeLaS3 cells^42^. Immunoprecipitation demonstrated a direct OPTN-NRF2 interaction, and we also found through immunofluorescence staining of endogenous OPTN and NRF2 that they were colocalized primarily in the perinuclear cytoplasm in preosteoclasts during osteoclastogenesis. At present, the nature of these perinuclear OPTN-NRF2 complexes is unclear, as is their role in regulating downstream NRF2-responsive antioxidant genes. These complexes might be involved in modulating protein transport, degradation, or activity. These possibilities were investigated, and our data suggested that NRF2 nuclear translocation was compromised. Neither NRF2 degradation nor transcriptional activity was affected in the *Optn*^*−/−*^ cells. Consistent with our results, *Nrf2* global knockout mice have been reported to exhibit augmented intracellular ROS, enhanced RANKL-induced osteoclastogenesis, and increased osteoclastic gene expression due to the impaired antioxidant response^28^. Future work will be required to elucidate the nature of the OPTN-NRF2 interactions and the mechanism of how this interaction regulates the antioxidant response.

It has been reported that osteoclasts in Nrf2 global knockout mice exhibit augmented intracellular ROS, enhanced RANKL-induced osteoclastogenesis, and increased osteoclastic gene expression due to the impairment of the antioxidant response^28^. In addition, our previous study identified the dual roles of the NRF2-mediated cellular adaptive response in RANKL-induced osteoclastogenesis, including the protective roles of cells against oxidative damage and the inhibitory role of RANKL-stimulated ROS signaling^54^. Based on these findings, we further evaluated the treatment effects of multiple compounds that can activate NRF2 in the *Optn*^*−/−*^ cells. Notably, all of these compounds showed a strong ability to attenuate the enhanced osteoclastogenesis in the *Optn*^*−/−*^ cells, suggesting that these types of NRF2 activators represent novel candidates for the prevention and treatment of diseases involving hyperactivated osteoclasts, such as PDB. Furthermore, these findings support our hypothesis that OPTN regulates osteoclast differentiation by mediating ROS signaling.

The mechanism by which osteoclastogenesis and bone loss increase with aging in vivo remains unclear. The genetic association of PDB with OPTN allowed the development of a genetic PDB mouse model to investigate this mechanism, which we have previously reported^36^. Although this model exhibited age-dependent bone degeneration consistent with PDB, it remains unclear whether deficiency of the antioxidant response plays a role in vivo. Because it has been reported that uncontrolled ROS will induce progressive cellular damage during the aging process^55^, we hypothesized that this accumulation of ROS with age and a concomitant decrease in antioxidants may result in elevated osteoclastogenesis, which ultimately causes the PDB-like phenotype in our *Optn*^*−/−*^ mouse model. However, given that the pathogenesis of PDB is clearly complex, multifactorial, and time-dependent, the underlying mechanisms by which OPTN plays a role in the process require further investigation.

While physiological levels of ROS can serve as secondary messengers in multiple signaling cascades^54,56,57^, excessive ROS lead to oxidative stress and, if not contained, will eventually damage lipids, proteins, and DNA, resulting in oxidative damage. Our data (Fig. 3) showed that the *Optn*^*−/−*^ cells are more susceptible to the cytotoxicity induced by high levels of H_2_O_2_ than the control cells, which is probably due to the attenuated antioxidative capacities of cells. This decrease in antioxidants and a concomitant accumulation of ROS with age could be a potential explanation for this age-dependent bone loss. Moreover, uncontrolled ROS accumulation with aging and the resulting oxidative damage have been implicated in various disorders, such as cancer, neurodegeneration, and other age-dependent diseases^58^. Our discovery that OPTN regulates ROS through NRF2-dependent antioxidants may have substantial translational and clinical significance beyond disorders of bone degeneration. OPTN has also been genetically associated with neurodegeneration in glaucoma and ALS^30–32^, suggesting that our findings may contribute to novel therapies for neurodegenerative diseases.

## Supplementary information

Supplementary data
