# Supplementary material for: Deficiency of optineurin enhances osteoclast differentiation by attenuating the NRF2-mediated antioxidant response
Source: Exp Mol Med. 2021 Apr 16;53(4):667–80. doi: 10.1038/s12276-021-00596-w (PMC8102640; doi:10.1038/s12276-021-00596-w)
Supplement: Supplementary file 1 — Supplementary data [file 12276_2021_596_MOESM1_ESM.pdf]

Table S1

Table S1. Primer sequences used for real-time quantitative RT-PCR

Target cDNA	Forward sequence (5'-3')	Reverse sequence (5'-3')
β-actin	GGCTGTATTCCCCTCCATCG	CCAGTTGGTAACAATGCCATGT
Ho1	AAGCCGAGAATGCTGAGTTCA	GCCGTGTAGATATGGTACAAGGA
Nqo1	AGGATGGGAGGTACTCGAATC	AGGCGTCCTTCCTTATATGCTA
Gclc	GGGGTGACGAGGTGGAGTA	GTTGGGGTTTGTCCTCTCCC
Gclm	AGGAGCTTCGGGACTGTATCC	GGGACATGGTGCATTCCAAAA
Srx	ATCGTGGTGCTGGATTGATTC	CACCCCAGAGATAAGATTACCCA
Nfatc1	GACCCGGAGTTCGACTTCG	TGACACTAGGGGACACATAACTG
c-Fos	CGGGTTTCAACGCCGACTA	TTGGCACTAGAGACGGACAGA
Trap	AGCAGCCAAGGAGGACTACGTT	TCGTTGATGTCGCACAGAGG
Ctsk	AGGGAAGCAAGCACTGGATA	GCTGGCTGGAATCACATCTT

Figure S1

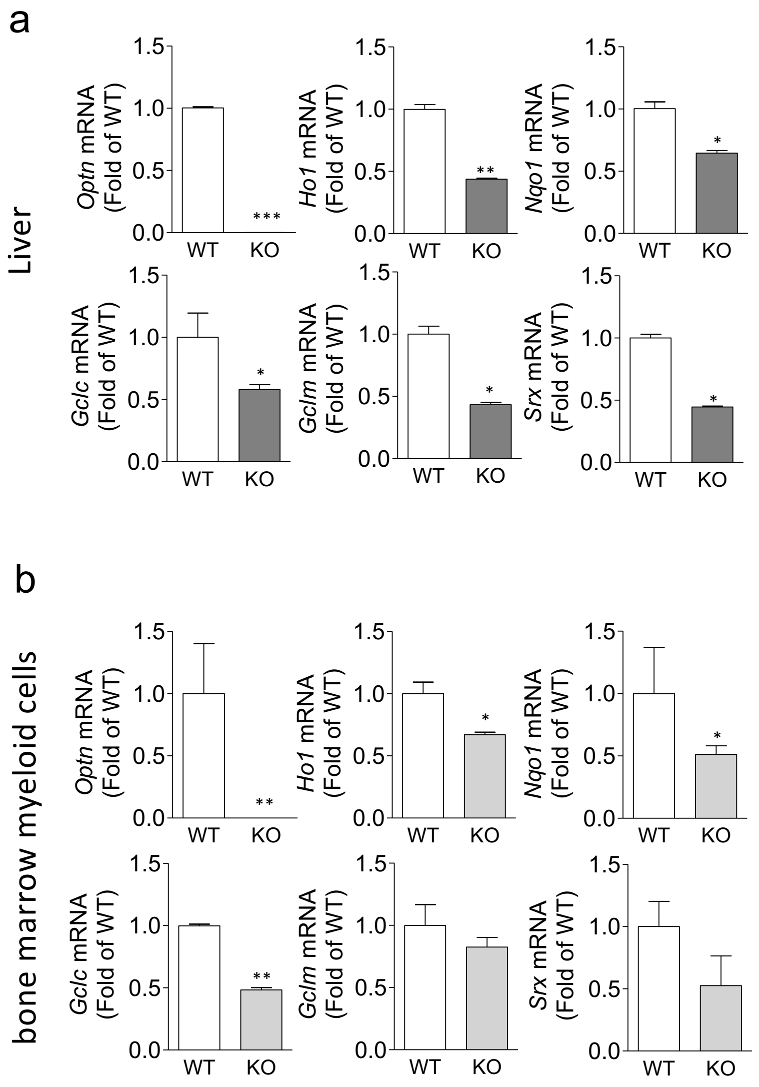
a
Liver
Optn mRNA (Fold of WT)
Ho1 mRNA (Fold of WT)
Nqo1 mRNA (Fold of WT)
Gclc mRNA (Fold of WT)
Gclm mRNA (Fold of WT)
Srx mRNA (Fold of WT)
0.0
0.5
1.0
1.5
WT
KO
***
**
*
b
bone marrow myeloid cells

Figure S2

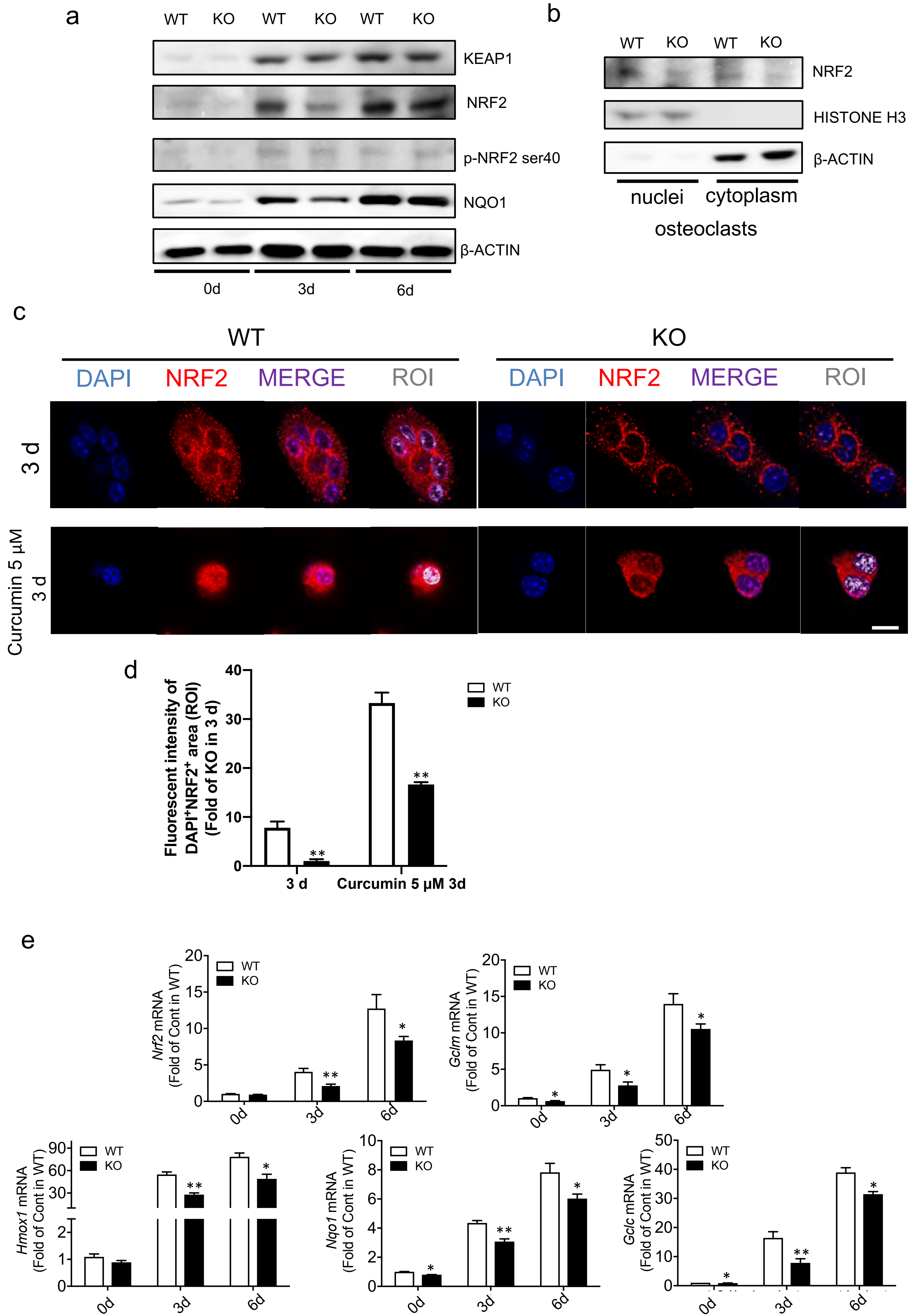
a
WT KO WT KO WT KO
KEAP1
NRF2
p-NRF2 ser40
NQO1
β-ACTIN
0d
3d
6d
b
WT KO WT KO
NRF2
HISTONE H3
β-ACTIN
nuclei
cytoplasm
osteoclasts
c
WT
KO
DAPI
NRF2
MERGE
ROI
3 d
Curcumin 5 μM
3 d
d
Fluorescent intensity of DAPI+NRF2+ area (ROI) (Fold of KO in 3 d)
0
10
20
30
40
WT
KO
**
**
3 d
Curcumin 5 μM 3d
e
Nrf2 mRNA (Fold of Cont in WT)
Gclm mRNA (Fold of Cont in WT)
Hmox1 mRNA (Fold of Cont in WT)
Nqo1 mRNA (Fold of Cont in WT)
Gclc mRNA (Fold of Cont in WT)
WT
KO
0d
3d
6d
*
**

Figure S3

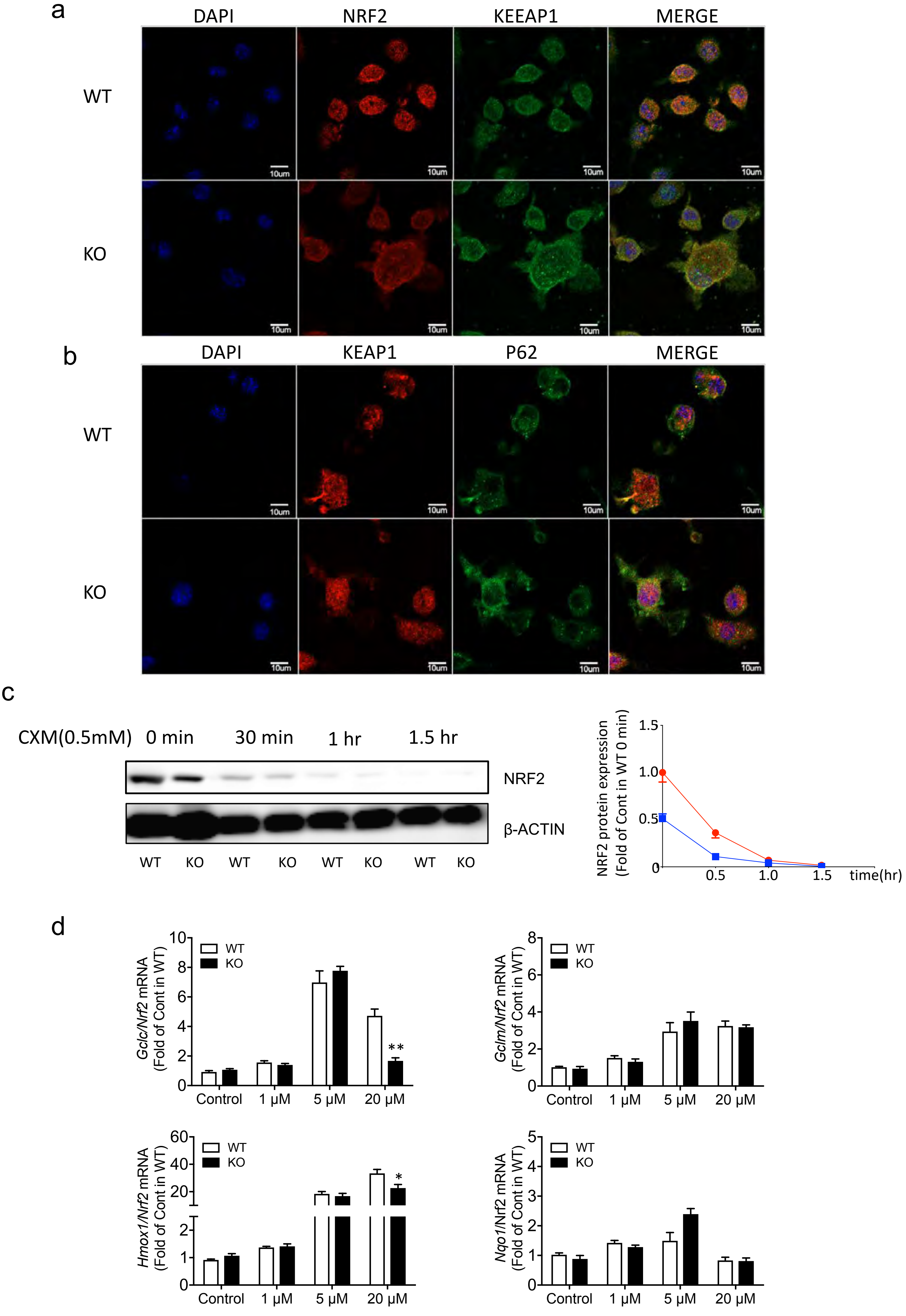
a
DAPI
NRF2
KEEAP1
MERGE
WT
KO
10um
b
DAPI
KEAP1
P62
MERGE
WT
KO
10um
c
CXM(0.5mM)
0 min
30 min
1 hr
1.5 hr
NRF2
β-ACTIN
WT
KO
NRF2 protein expression
(Fold of Cont in WT 0 min)
1.5
1.0
0.5
0
0.5
1.0
1.5
time(hr)
d
Gclc/Nrf2 mRNA
(Fold of Cont in WT)
Gclm/Nrf2 mRNA
(Fold of Cont in WT)
Hmox1/Nrf2 mRNA
(Fold of Cont in WT)
Nqo1/Nrf2 mRNA
(Fold of Cont in WT)
WT
KO
Control
1 μM
5 μM
20 μM
**
*
10
8
6
4
2
0
60
40
20
5
3
1

Figure S4

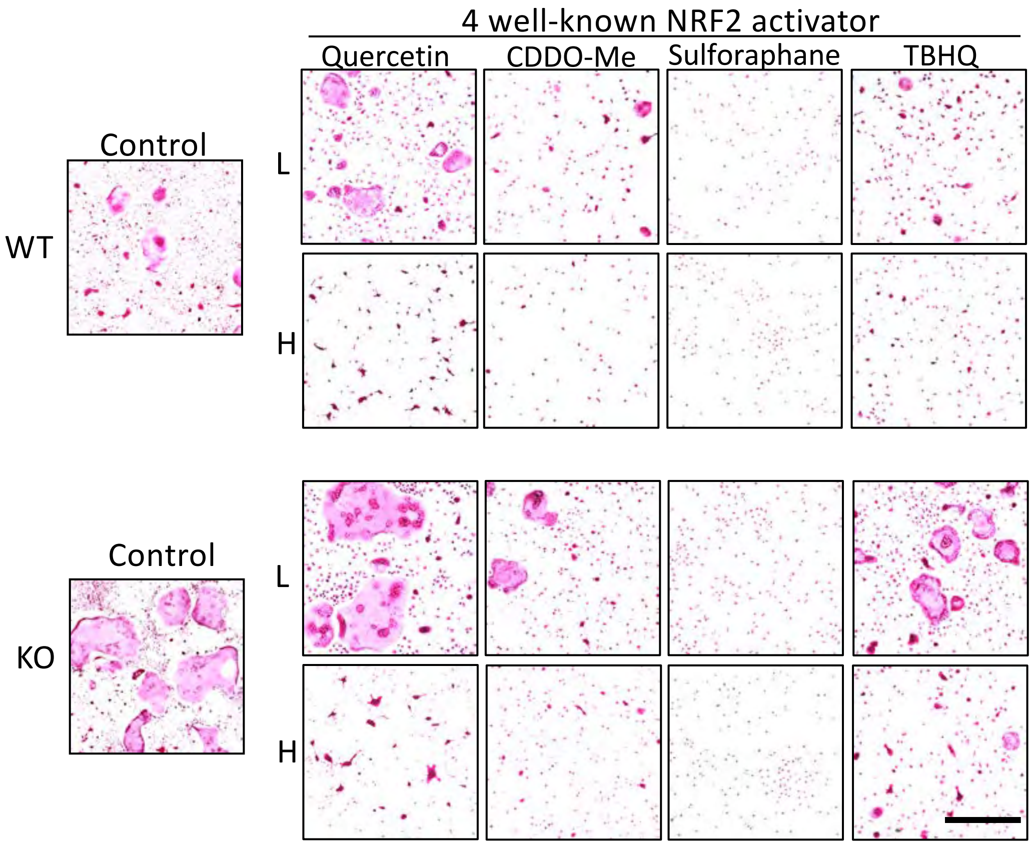
4 well-known NRF2 activator
Quercetin
CDDO-Me
Sulforaphane
TBHQ
Control
WT
L
H
Control
KO
L
H
